# Interactive effects of gallic/ferulic/caffeic acids and anthocyanins on pigment thermal stabilities

**DOI:** 10.1016/j.dib.2017.04.036

**Published:** 2017-04-28

**Authors:** Bing-Jun Qian, Jian-Hua Liu, Shu-Juan Zhao, Jian-Xiong Cai, Pu Jing

**Affiliations:** a Research Center for Food Safety and Nutrition, Key Lab of Urban Agriculture (South), Bor S. Luh Food Safety Research Center, School of Agriculture & Biology, Shanghai Jiao Tong University, Shanghai 200240, China; b College of Resources and Environment Engineering, Yibin University, Yibin, Sichuan 644000, China

**Keywords:** Purple sweet potato, Anthocyanin, Phenolic acid, Color, Molecular dynamic simulation

## Abstract

The data presented in this article are related to the research article entitled “The effects of gallic/ferulic/caffeic acids on colour intensification and anthocyanin stability” (Qian et al., 2017) [1]. This paper described preparation and isolation of anthocyanins from purple sweet potatoes (PSP) and the time-course of anthocyanin profiles treated with gallic, ferulic, or caffeic acids at 95 °C. The color appearance of PSPanthocyanins alone, or with gallic, ferulic, or caffeic acids was described after the 15 h of thermal treatment. The high resolution mass spectrographs of PSP anthocyanins were determined using UPLC-ESI-HRMS. The spatial interaction of peonidin 3-O-(2-O-β-D-glucopyranocyl-β-D-glucopyranoide)-5-O-β-D-glucopyranoside and gallic/ferulic/caffeic acids was illustrated by molecular dynamic simulation.

**Specifications Table**

**Table:** 

Subject area	*Chemistry*
More specific subject area	*Pigments and food colorants*
Type of data	*Figure*
How data was acquired	*HPLC, mass spectroscopy, computer simulation*
Data format	*Raw data collection and analysis*
Experimental factors	*Thermal treatment*
Experimental features	*Experimental and theoretical studies. 3 replicates were used in the experiment as complete randomized design. Computationally analyzed.*
Data source location	*Shanghai, China*
Data accessibility	*The data is with this article.*

**Value of the data**

• Data of anthocyanin profiles during thermal treatment can be valuable to further study for stability of individual anthocyanin complexes with gallic, ferulic, or caffeic acids.
• The data provide a theoretically understanding the interaction of peonidin 3-*O*-(2-*O*-β-D-glucopyranocyl-β-D-glucopyranoide)-5-*O*-β-D-glucopyranoside with gallic, ferulic, or caffeic acids.
• Identification of the interaction of anthocyanins and other potential copigments serve as the paradigm for the researcher in further studies for pigments and food colorants.

## 1 Data

**Fig.1.1:**
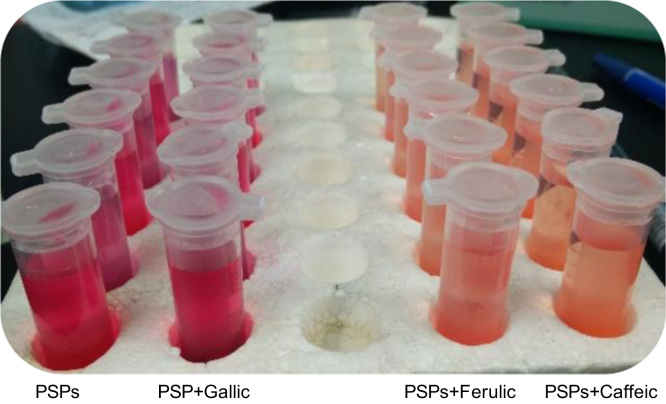
Samples of complexes of PSP anthocyanins with gallic, ferulic, or caffeic acids at 95 °C for 15 h.

**Fig. 1.2:**
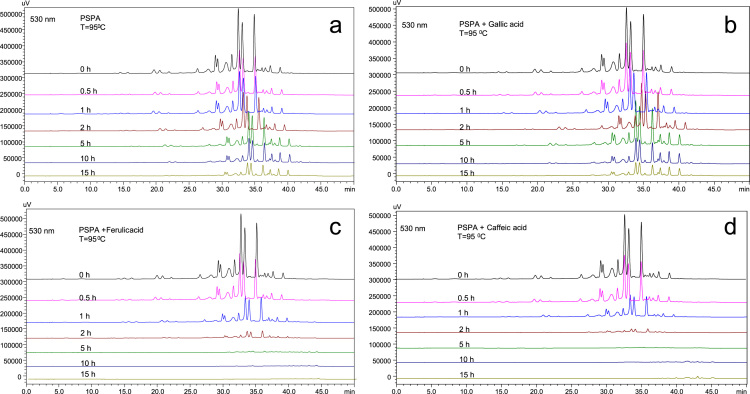
HPLC profiles of PSP anthocyanins enhanced by three phenolic acids at 95 °C. (a) PSP anthocyanins; (b) PSP anthocyanins with ferulic acids; (c) PSP anthocyanins with gallic acids; (d) PSP anthocyanins with caffeic acids.

**Fig. 1.3:**
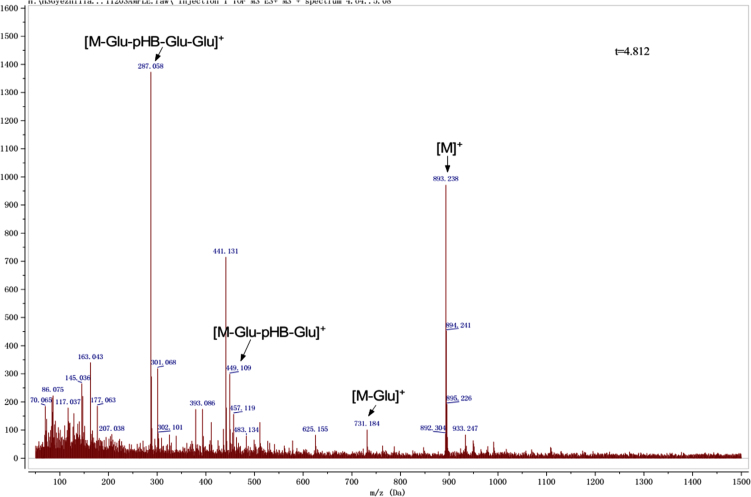
MS spectra of cyanidin-3-*p*-hydroxybenzoyl sophoroside-5-glucoside.

**Fig. 1.18:**
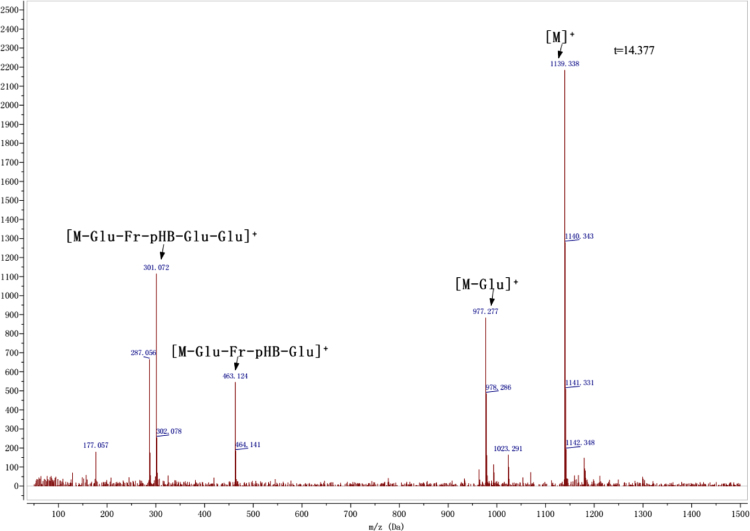
MS spectra of peonidin-3-(6″, 6″′-diferuloyl sophoroside)-5-glucoside.

**Fig.1.19:**
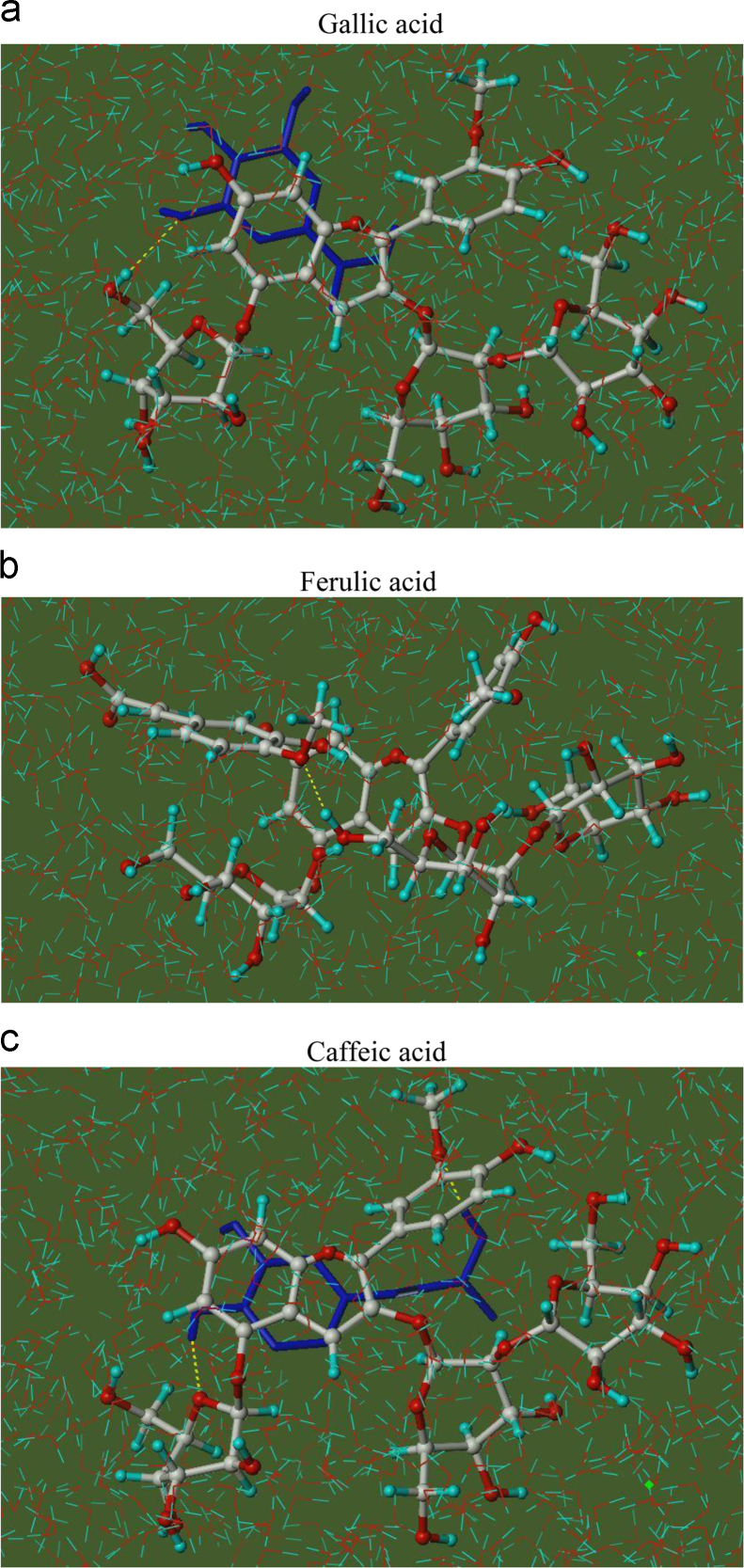
Complexes of peonidin 3-*O*-(2-*O*-β-D-glucopyranocyl-β-D-glucopyranoide)-5-*O*-β-D-glucopyranoside with gallic acid (a), ferulic acid (b), and caffeic acid (c) with closest geometries to the average structures after molecular dynamics simulations at 368 K. (a) Gallic acid, (b) ferulic acid, and (c) caffeic acid.

Anthocyanins in complexes with gallic/ferulic/caffeic acids were evaluated using an accelerated stability test at 95 °C, and sampled at regular intervals (0, 0.5, 1, 2, 5, 10, and 15 h). Fig. 1.1 shows the color appearance of anthocyanin complexes collected at 15 h. Fig. 1.2 shows the dynamic variation in anthocyanins added with gallic/ferulic/caffeic acids by HPLC profiles. Fig. 1.2 shows the remaining anthocyanins during thermal treatment at 95 °C. Fig. 1.2b–d shows the remaining anthocyanins with gallic, ferulic, or caffeic acids during thermal treatment at 95 °C. Anthocyanins in PSPs were identified *via* a high-resolution mass spectrometer in Figs. 1.3**–**1.18. Additionally, Fig. 1.19 shows molecular dynamics simulation for analysis of the copigmentation behavior of gallic (Fig. 1.19a), ferulic (Fig. 1.19b), and caffeic (Fig. 1.19c) acids over peonidin 3-*O*-(2-*O*-β-D-glucopyranocyl-β-D-glucopyranoide)-5-*O*-β-D-glucopyranoside, which was predominant in PSPs.

## 2 Experimental design, materials and methods

### 2.1 Extraction and purification of anthocyanins

Extraction and purification of PSP anthocyanins were executed following the previous study [2]. Fresh PSPs (5 kg) were crushed into puree, followed by being immersed in 10 L of methanol containing 0.01% HCl and extracted for 2 h. The raw extracts were applied to a 600 cm×50 cm Amberlite XAD-7HP column (Huideyi, Beijing, China) and washed with 0.01% aqueous HCl to remove water-soluble compounds. The anthocyanin fraction was eluted with 0.01% ethanolic HCl and applied to a 100 cm×2.5 cm Sephadex LH-20 column and separated by 50% aqueous ethanol containing 0.01% HCl. Monomeric anthocyanin isolation was achieved in an Agilent preparative HPLC system. Mobile phases were (A) water containing 0.1% formic acid (v/v) and (B) methanol containing 0.1% formic acid (v/v). Separation was achieved with the following gradient program: 30% B, 0–3 min; 50% B, 3–10 min; 70% B, 10–12 min. An injection volume of 10 mL with a 5 mL/min flow rate was used. The peak fraction was collected and dried with a nitrogen-blow evaporator. Purity of the anthocyanins obtained was more than 30% (w/w).

### 2.2 Thermal stability of copigmentation

The pigment stability of PSP anthocyanins was evaluated using an accelerated stability test at 95 °C [1]. The pH 3.2 buffer (0.06 mol/L sodium acetate and 0.02 mol/L phosphoric acid) was used to prepare pigment solutions, which contained 22.46 mg monomeric anthocyanins/L of PSP anthocyanin extract alone or with additional gallic acid (851 mg), ferulic acid (971 mg), or caffeic acid (901 mg) to satisfy the 1:100 M ratio of cyanidin-3-glucoside and phenolic acid. Ten microliter of each solution were added into test tubes which were then closed with screw caps. All tubes covered with aluminum foil were then immersed in a water bath at 95 °C. Twelve tubes were prepared and sampled at regular intervals (0, 0.5, 1, 2, 5, 10, and 15 h) and rapidly cooled to room temperature for analysis. Triplicates were performed for the thermal treatment.

### 2.3 Quantitative analysis of individual PSP anthocyanins

The monomeric anthocyanins in PSPs during thermal treatment was identified using a LC-2030C HPLC system (Shimadzu, Japan), which was equipped with a binary solvent delivery system, an online vacuum degasser, an automatic sampler, a thermostatically controlled column compartment and a diode array detection (PDA) system. Separation was executed on an InertSustain C_18_ column (5 μm, 250 mm×4.6 mm i.d.) by a reverse phase elution at a flow rate of 1 mL/min. The injection volume was 20 µL. Formic acid (1%, v/v) in water or acetonitrile was used as the mobile phases A or B, respectively. The gradient program was as follows: 0–5 min, 10% B; 5–20 min, 10–15% B; 20–30 min, 15–20% B; 30–40 min, 20–25%B; 40–45 min, 25–40% B; 45–50 min, 40–60% B. Spectral information was collected over the wavelength range from 200 nm to 800 nm, where the total absorbance of anthocyanins was recorded at 530 nm. The total absorbance of anthocyanins was analyzed using a linear regression analysis. Samples from each thermal treatment were analyzed in triplicate.

### 2.4 UPLC-ESI-HRMS analysis of PSP anthocyanins

Separation of the anthocyanins were finished using an ACQUITY UPLC system (Waters, MA, USA) equipped with an Acquity BEH C_18_ column (1.7 μm, 100 mm×2.1 mm i.d., Waters, MA, USA). Mobile phase A was 0.1% formic acid in water (v/v), and mobile phase B was acetonitrile. The anthocyanins were eluted with a linear gradient of mobile phase B in A going from 5% to 20% over 15 min, from 20% to 40% over 5 min and from 40% to 85% over 2 min at a flow rate of 0.4 mL/min. Spectral information was collected over the wavelength range from 200 to 800 nm.

The Waters Micromass Q-TOF Premier mass spectrometer equipped with an electrospray interface (Waters, MA, USA) was used to detect anthocyanins. The positive ionization mode was applied with the electrospray/ion optics parameters set as follows: capillary voltage, 3.0 kV; sampling cone, 35 V; collision energy, 4 eV; source temperature, 115 °C; desolvation temperature, 250 °C; desolvation gas flow, 300 L/h. The scan time was 0.5 s over an *m/z* range from 50 to 1500 au.

### 2.5 Molecular dynamics simulation

The initial geometries of peonidin 3-O-(2-O-β-D-glucopyranocyl-β-d-glucopyranoide)-5-O-β-d-glucopyranoside (P3GG5G) and the phenolic acids (caffeic, ferulic, and gallic acids) were built using the SYBYL X-1.3 software on a Windows operating system. The Powell method was applied to minimize energy of each structure using the Tripos force field, and the maximum iterations were set to 1000 steps and other default parameters was set according to our previous work [3]. The Powell conjugate gradient algorithm was terminated until the convergence criterion of 0.005 kal/(mol Å) was satisfied. The partial atomic charges were calculated using the Gasteiger-Hücke method. Molecular docking was performed by the Surflex-dock model, where the lowest-energy conformations of peonidin-3-sophoroside-5-glucoside as the receptor and the phenolic acid as the ligand were used. The molecules of water and bound ligands were removed from the receptor prior to docking analysis. All parameters were default, and CScore calculation was off. All the putative docking models were analyzed and selected after docking completion based on the MolDock score and hydrogen bonding interactions. And then the best docked complexes were further analyzed by molecular dynamics (MD) simulations. MD simulations in periodic boundary conditions were performed using the Tripos force field with the silverware algorithm for solvation. The positive charge of each studied system was neutralized with one counterion (Cl^−^). Each complex was minimized in two stages: the complexes were fixed, and only the water and position of the counterion were minimized; the full system was minimized. Subsequently, the whole system was equilibrated by 100 ps MD, followed by a 10 ns MD simulation using a fixed pressure P, temperature T, and number of atoms N (constant-NPT ensemble) at 368 K with 50 fs of integration time and a cut-off of 10 angstroms for long-range interactions. A 31 Å × 31  Å ×31 Å box around the water was applied to simulate the periodic boundary conditions. The final modeled system contained 1393 P3GG5G, phenolic acid, and water together. The stabilized structure was applied to determine the possibility to form hydrogen bonds (H bonding ability), which were also visualized with the sites and number of direct and intermediate hydrogen bonds [4].

### 2.6 Statistical analysis

A completely randomized design (CRD) was used to design the accelerated stability test with three replicates for each treatment.

## Figures and Tables

**Fig. 1.4:**
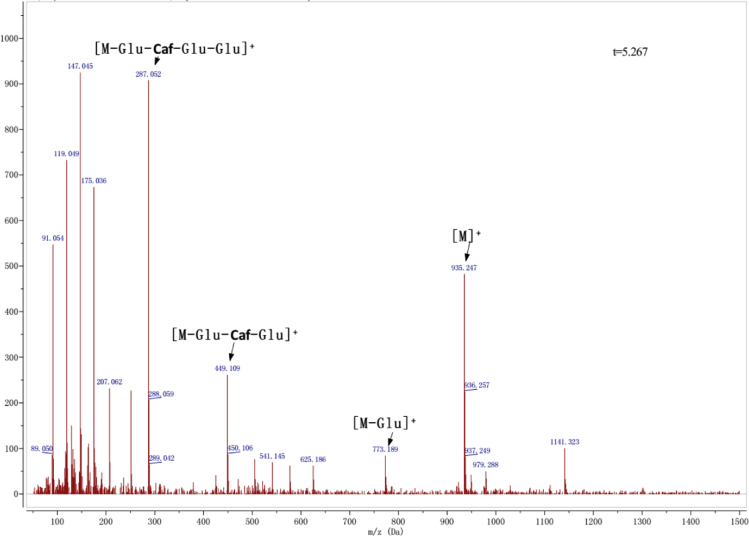
MS spectra of cyanidin-3-(6′′-caffeoyl sophoroside)-5-glucoside.

**Fig. 1.5:**
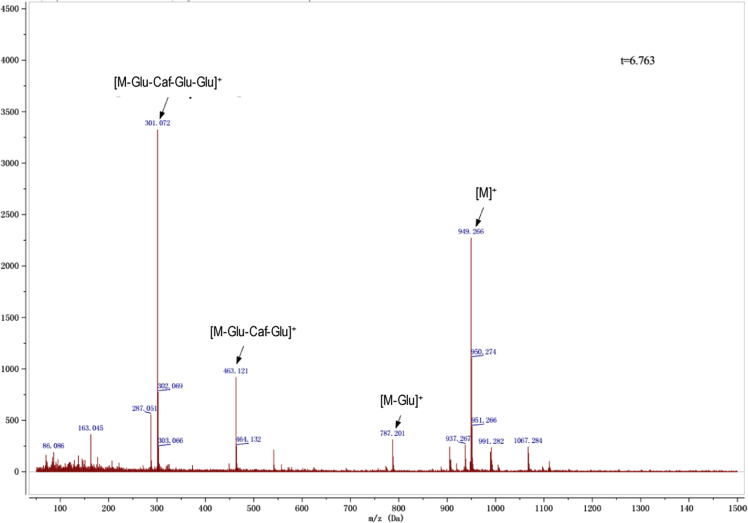
MS spectra of peonidin-3-*p*-hydroxybenzoyl sophoroside-5-glucoside.

**Fig. 1.6:**
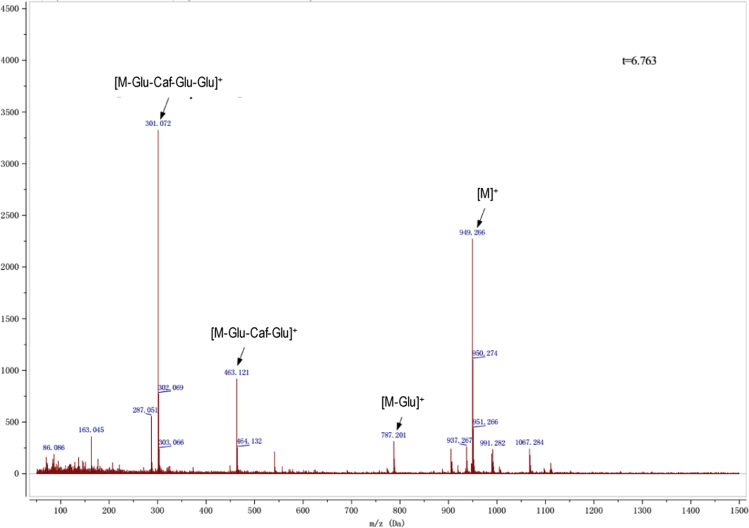
MS spectra of peonidin-3-caffeoyl sophoroside-5-glucoside.

**Fig. 1.7:**
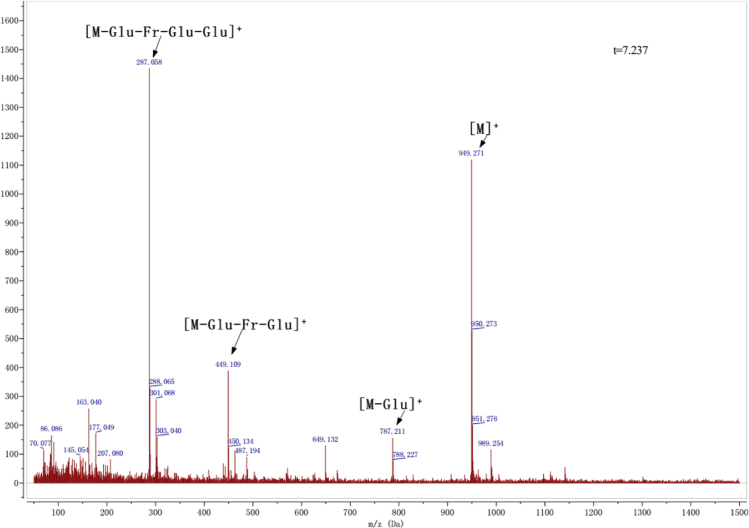
MS spectra of cyanidin-3-(6″-feruloyl sophoroside)-5-glucoside.

**Fig. 1.8:**
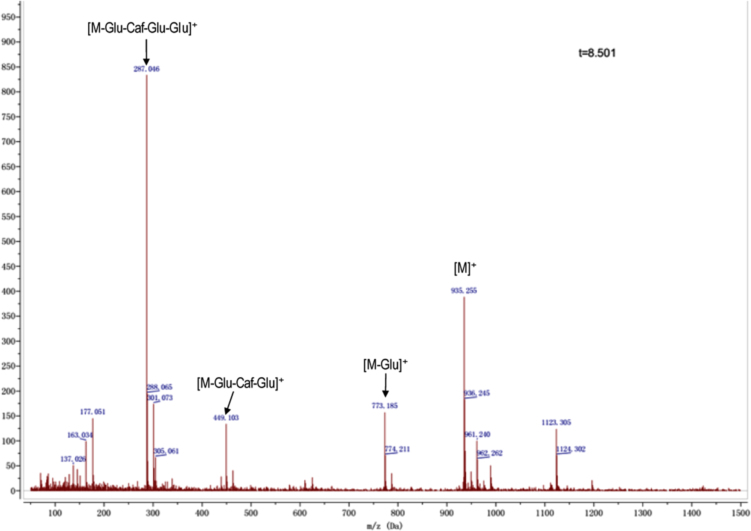
MS spectra of cyanidin-3-caffeoyl glucoside-5-glucoside.

**Fig. 1.9:**
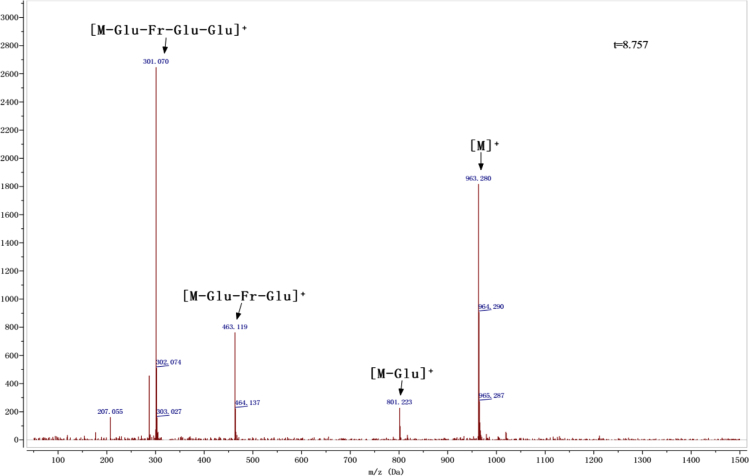
MS spectra of peonidin-3-(6″-feruloyl sophoroside)-5-glucoside.

**Fig. 1.10:**
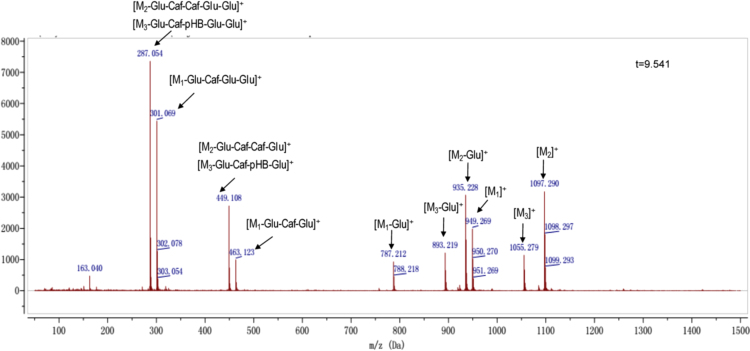
MS spectra of peonidin-3-caffeoyl sophoroside-5-glucoside isomer (M_1_), cyanidin 3-(6″, 6″′-dicaffeoyl sophoroside)-5-glucoside (M_2_), and cyanidin-3-caffeoyl-*p*-hydroxybenzoyl sophoroside-5-glucoside (M_3_).

**Fig. 1.11:**
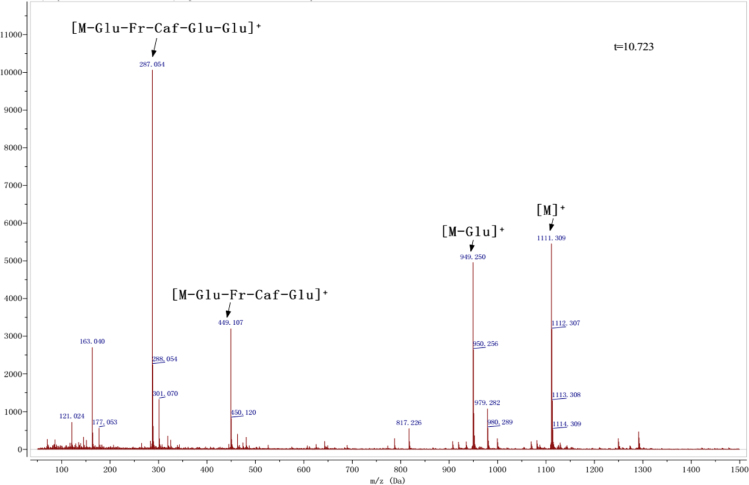
MS spectra of cyanidin-3-(6″-caffeoyl-6″′-feruloyl sophoroside)-5-glucoside.

**Fig. 1.12:**
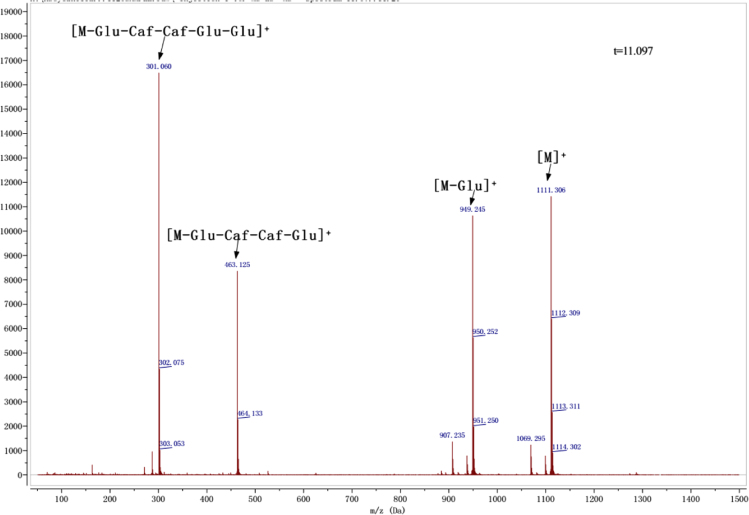
MS spectra of peonidin-3-(6″, 6″′-dicaffeoyl sophoroside)-5-glucoside.

**Fig. 1.13:**
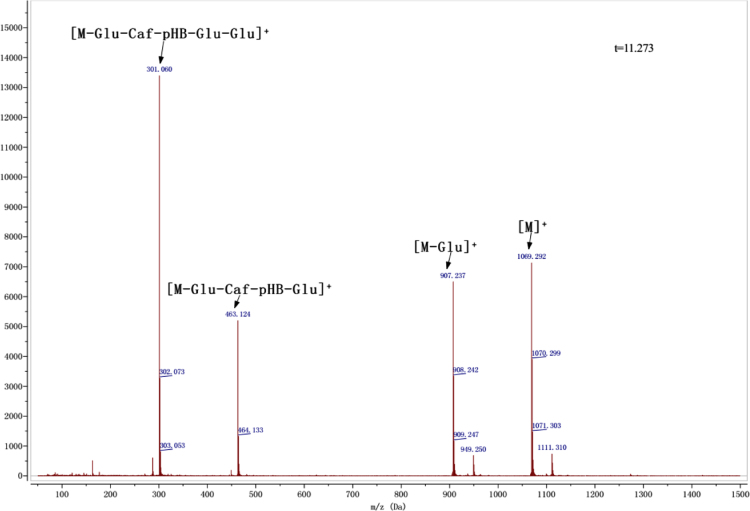
MS spectra of peonidin-3-caffeoyl *p-*hydroxybenzoyl-sophoroside-5-glucoside.

**Fig. 1.14:**
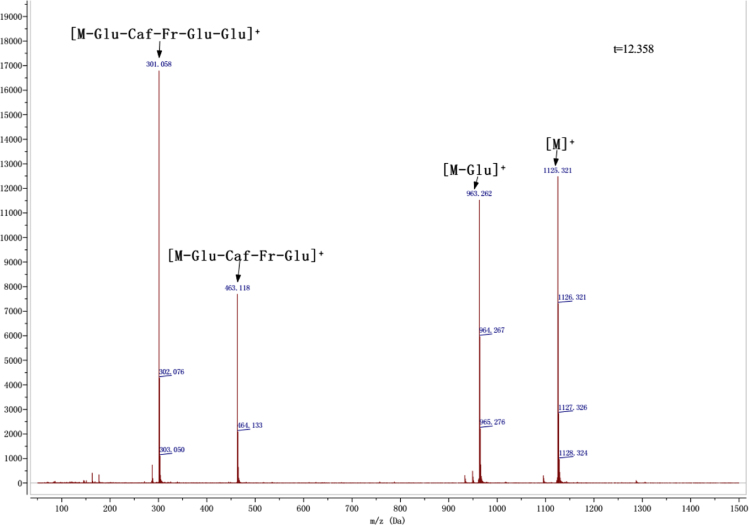
MS spectra of peonidin-3-(6″-caffeoyl-6″′-feruloyl sophoroside)-5-glucoside.

**Fig. 1.15:**
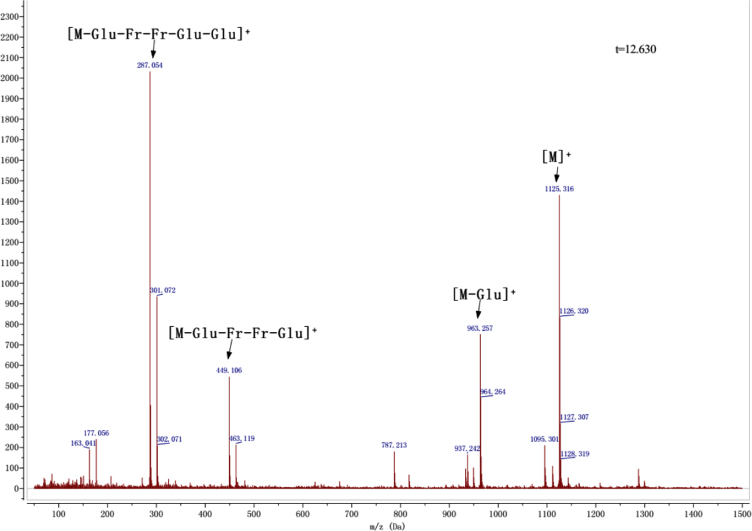
MS spectra of cyanidin 3-(6″, 6″′-diferuloyl sophoroside)-5-glucoside.

**Fig. 1.16:**
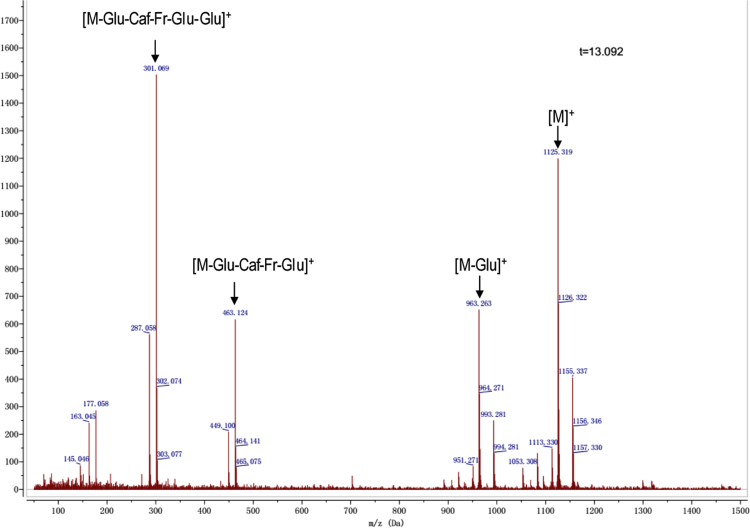
MS spectra of penodin-3-caffeoyl feruloyl sophoroside-5-glucoside isomer.

**Fig. 1.17:**
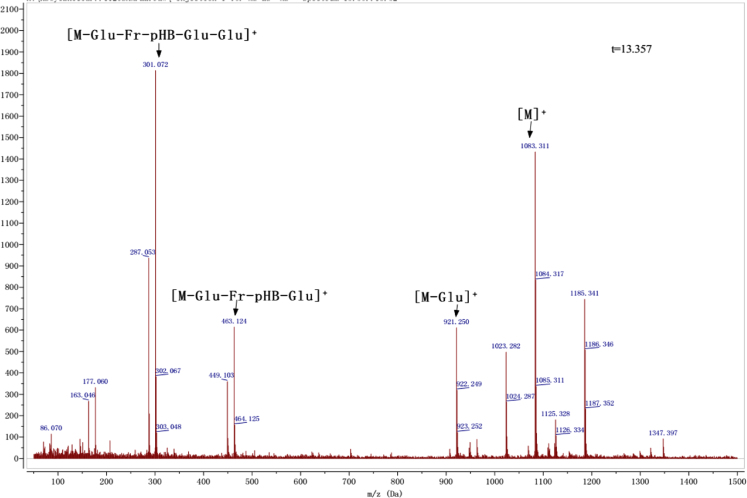
MS spectra of peonidin 3-feruloyl-p-hydroxy benzoyl sophoroside-5-glucoside.
